# Gene Regulation of Pteridine Reductase 1 in *Leishmania* Promastigotes and Amastigotes Using a Full-Length Antisense Construct

**Published:** 2013

**Authors:** F Kheirandish, M Bandehpour, N Davoudi, N Mosaffa, S Dawood, B Kazemi, A Haghighi, A Khamesipour, H Masjedi, M Mohebali, F Mahboudi

**Affiliations:** 1 Department of Parasitology and Mycology, School of Medicine, Lorestan University of Medical Sciences, Khorramabad, Iran; 2 Cellular and Molecular Biology Research Center, Shahid Beheshti University of Medical Sciences., Tehran, Iran; 3 Department of Biotechnology, Institute Pasteur of Iran, Tehran, Iran; 4 Department of Immunology, School of Medicine, Shahid Beheshti University of Medical Sciences, Tehran, Iran; 5 Skin Disease Hospital, Damascus University, Damascus, Syria; 6 Department of Parasitology and Mycology, School of Medicine, Shahid Beheshti University of Medical Sciences, Tehran, Iran; 7 Department of Biotechnology, School of Medicine, Shahid Beheshti University of Medical Sciences, Tehran, Iran; 8 Center for Research and Training in Skin Disease and Leprosy, Tehran University of Medical Sciences, Tehran, Iran; 9 Department of Parasitology and Mycology, School of Public Health, Tehran University of Medical Sciences, Tehran, Iran

**Keywords:** Pteridine reductase 1, Antisense, *Leishmania*, Gene regulation, Inhibition

## Abstract

**Background:**

Pteridine metabolic pathway is unusual features of *Leishmania*, which is necessary for the growth of parasite. *Leishmania* has evolved a complex and versatile pteridine salvage network which has the ability of scavenging a wide area of the conjugated and unconjugated pteridines especially folate and biopterin. In this study, we focus on the inhibition of *ptr1* gene expression.

**Methods:**

*L. major ptr1* gene was cloned into pcDNA3 and digested using KpnI and BamHI. The gene was subcloned so that antisense will transcribe and called pcDNA-rPTR. *Leishmania major* was cultured and late logarithmic-phase promastigotes were harvested. The promastigotes were divided into two groups. One group was transfected with 50 µg of pcDNA-rPTR, whereas the other group was transfected with pcDNA3. Transfected cells were cultured and plated onto semi-solid media. Mouse pritonean macrophages were transfected using pcDNA-rPTR–tansfected promastigotes. Western blotting was performed on mouse transfected pritonean macrophages and extracts from transfected promastigotes of *L. major* using a *L. major ptr1* antibody raised in rabbits.

**Results:**

The PTR1 protein was not expressed in pcDNA-rPTR– tansfected promastigotes and mouse macrophage transfected with pcDNA-rPTR– tansfected promastigotes.

**Conclusion:**

This approach might be used to study the pteridine salvage pathway in *Leishmania* or to assess the possibility of using gene expression inhibition in the treatment of leishmaniasis.

## Introduction

Pteridine metabolic pathway is unusual features of *Leishmania*, which is necessary for the growth of parasite. Different parts of pteridine pathway are the target of chemotherapeutic. In order to infect the mammalian host cells pteridine auxotroph, *Leishmania* absolutely needs an exogenous source of pterin (1–3). *Leishmania* has evolved a complex and versatile pteridine salvage network which has the ability of scavenging a wide area of the conjugated and unconjugated pteridines especially folate and biopterin (4). Folate and biopterin in fully reduced tetrahydro forms, H4-folate and H4-biopterin, act as co-factors. In *Leishmania* and mammalian cells, NADPH-dependent enzyme dihydrofolate reductase (DHFR) is the agent for the production of H4-folate from folate and dihydrofolate (5). In *Leishmania* and other protozoa, DHFR exists as a bifunctional enzyme and joines to thymidylate synthase (DHFR-TS) (6). In the de novo biosynthesis of thymidylate in *Leishmania*, the main role of H4-folate is as essential co-factor (7).

In mammalian cells, H4-biopterin is synthesized through de novo or salvaged through DHFR-mediated reduction of H2-biopterin (8). While *Leishmania* lacks de novo biopterin synthetic pathway, DHFR-TS shows no response to biopterin or H2biopterin. Instead, reduced biopterin is generated through the pteridine reductase 1 (*ptr1*), which sequentially reduces oxidized biopterin to H2-biopterin and then to H4-biopterin (2, 3, 9, 10). *Ptr1* is as a gene within the *Leishmania* H region. Over exposure of *ptr1* by gene amplification or DNA transfixion, confers methotrexate (MTX) resistance (3, 11). The predicted *ptr1* protein showed homology to a large family of aldo/keto reductases and short-chain dehydrogenases, including several enzymes involved in pteridine metabolism, such as sepiapterin reductase (3) and dihydropteridine reductase (DHPR) (12). *Leishmania* needs reduced form of folate and biopterin for growth where as the current available anti-pteridines do not have much promising results clinically against leishmaniasis, even though were proved to be effective against other protozoan infectious which justifies research in this field (13).

In this study, *ptr1* gene expression was inhibited to study the pteridine salvage pathway in *Leishmania* or to assess the possibility of using gene expression inhibition in the treatment of leishmaniasis.

## Materials and Methods

### DNA extraction and gene amplification

*Leishmania major* was grown in NNN medium and subcultured in RPMI1640 enriched with 10% fetal bovine serum. DNA extraction was done on *Leishmania* promastigotes harvested at late logarithmic phase. A set of primers (PTR F, 5′-GGA TCC ATG ACT GCT CCG ACC-3′; PTR R, 5′-GGT ACC TCA GGC CCG GGT AAG-3′) was designed based on *L. major ptr1* sequence (GenBank Accession No. L01699) with BamHI and KpnI restriction sites established on the 5′-ends of the forward and reverse primers, respectively. The *ptr1* coding region was amplified from genomic DNA and the PCR product was ligated to a 3′ T-tailed, EcoRV-digested pBluescript and sequenced.

### Construction of the antisense ptr1 gene

Recombinant pBluescript containing *L. major ptr1* gene (accession no. EF113119) was digested with KpnI and BamHI enzymes and purified using a Fermentas DNA purification kit (Cat. No. k0513) and then subcloned into pcDNA3 digested with KpnI and BamHI. The recombinant plasmid was transformed into *E. coli* TOP10 strain. Since *ptr1* gene was cloned antiparallel to the sense, this expression plasmid is referred to pcDNA-rPTR or antisense.

### Transfection of Leishmania promastigotes

*Leishmania major* was cultured in liquid medium 199 (Sigma, UK, Dorset, England) supplemented with 10% defined heat-inactivated fetal bovine serum (Biosera, France), 10 mM adenine (Sigma, UK, Dorset, England), 40 mM HEPES (Sigma, UK, Dorset, England), 0.25% hemin (Sigma, UK, Dorset, England), 100 mg/ml streptomycin (Biosera, France) and 100 IU/ ml penicillin (Biosera, France). Late logarithmic phase *L. major* promastigotes were harvested by centrifugation at 1500*g* for 10 min and adjusted to 5 × 10^7^/ml in ice-cold transfection buffer (14–17). The promastigotes were divided into two groups: one group was transfected with 50 µg of pcDNA-rPTR (antisense), whereas the other group was transfected with only pcDNA3 using a BioRad Gene Pulser at 450 V and 450 µF capacitance as described previously (15–17). Transfected promastigotes were cultured for 48 h in drug-free medium 199 and subsequently plated onto semi-solid medium 199 containing 40 µg/ml G418 (Sigma, UK, Dorset, England) as a selective antibiotic because pcDNA3 was containing the neomycin resistance gene therefore this antibiotic was used to determine the transfection (14, 18).

### Inhibition of ptr1 gene expression in transfected promastigotes by antisense

After two weeks, single colonies were transferred into medium 199 supplemented with 40 µg/mL neomycin (14, 18). Transfected promastigotes were mass cultured and collected by centrifugation at 3,000 g for 10 min and washed twice using 1x TBS (150 mM NaCl, 10 mM Tris pH 7.5). Cells were resuspended in lysis buffer (50 mM Tris, 10% glycerol, 0.1% Triton X-100, 1 mM PMSF) and sonicated, then the lysate was centrifuged at 1,000 g for 10 min and the supernatant was used for analysis. Western blot analysis was performed as described previously (19). Briefly, 10^6^ of each pcDNA-rPTR- transfected *L. major* promastigotes and pcDNA3-transfected *L. major* promastigotes were harvested and lysed using sonication. Protein samples were separated on 10% SDS-PAGE, transferred to nitrocellulose membranes and incubated at 37 °C in the presence of a 1:500 dilution of rabbit anti-PTR1 antibody (20) for 1 hour. Then, the membrane was incubated at 37 °C for one hour using horseradish peroxidase (HRP)-conjugated goat anti-rabbit IgG (1:5000) as the secondary antibody. Antibody binding was visualized using diaminobenzidine (DAB) (19).

### Inhibition of ptr1 gene expression in amastigotes containing pcDNA-rPTR (antisense)

Macrophages were isolated from 4-6 weeks BALB/c mice by peritoneal lavage using chilled RPMI 1640 media (Biosera, France) containing 10% fetal bovine serum and allowed the macrophages to adhere to the surface of flasks for overnight incubation at 37°C in humidified 5% CO2. No adherent macrophages were washed off using Hanks solution. Adherent macrophages were incubated separately with two groups of transfectant parasites (pcDNA-rPTR- transfected *L. major* promastigotes and pcDNA3-transfected *L. major* promastigotes) and incubated at 37 °C in humidified 5% CO2 for 2 hours. Unbound parasites were removed by washing with Hanks solution and added RPMI1640 medium containing 10% fetal bovine serum and incubated at 37°C in humidified 5% CO2 for 3 days and the medium was changed every 24 h. Transfected macrophages were separated by scraper and harvested by centrifugation and then lysed by lysis buffer was added and incubated on ice for 30 min. SDS-PAGE and Western blot analysis was performed as described previously (19). Briefly, cells lysate (protein samples) were prepared and separated on 10% SDS-PAGE and electrophoretically transferred onto nitrocellulose membrane and analyzed using a rabbit anti-PTR1 antibody at a 1:1000 dilution as the primary antibody and Goat anti-rabbit IgG HRP conjugation at a 1:5000 dilution as secondary antibody and detected by colorimetry using diamino benzoic acid and H2O2.

## Results

### Construction of the antisense ptr1 gene

**Fig. 1:**
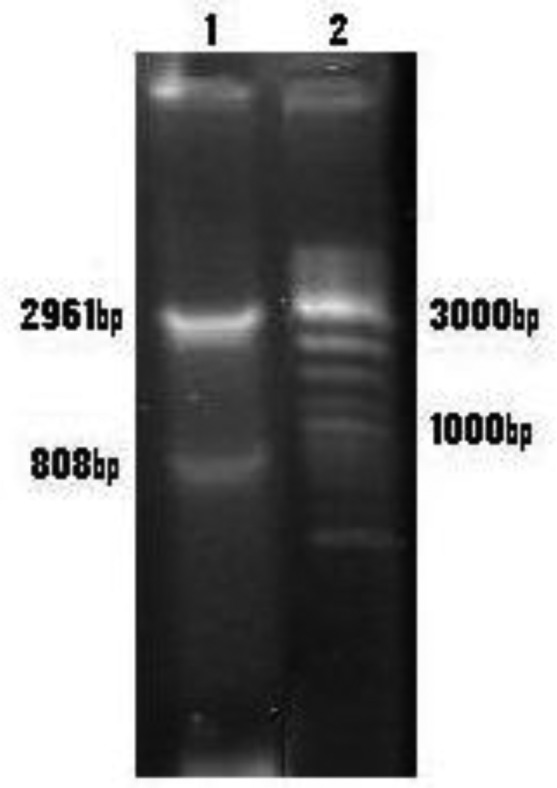
Electrophoresis of digested pBSC-ptr using enzyme/ Lane 1: Digested pBSC-ptr by KpnI and BamHI/Lane 2: 100-bp DNA ladder marker

**Fig. 2:**
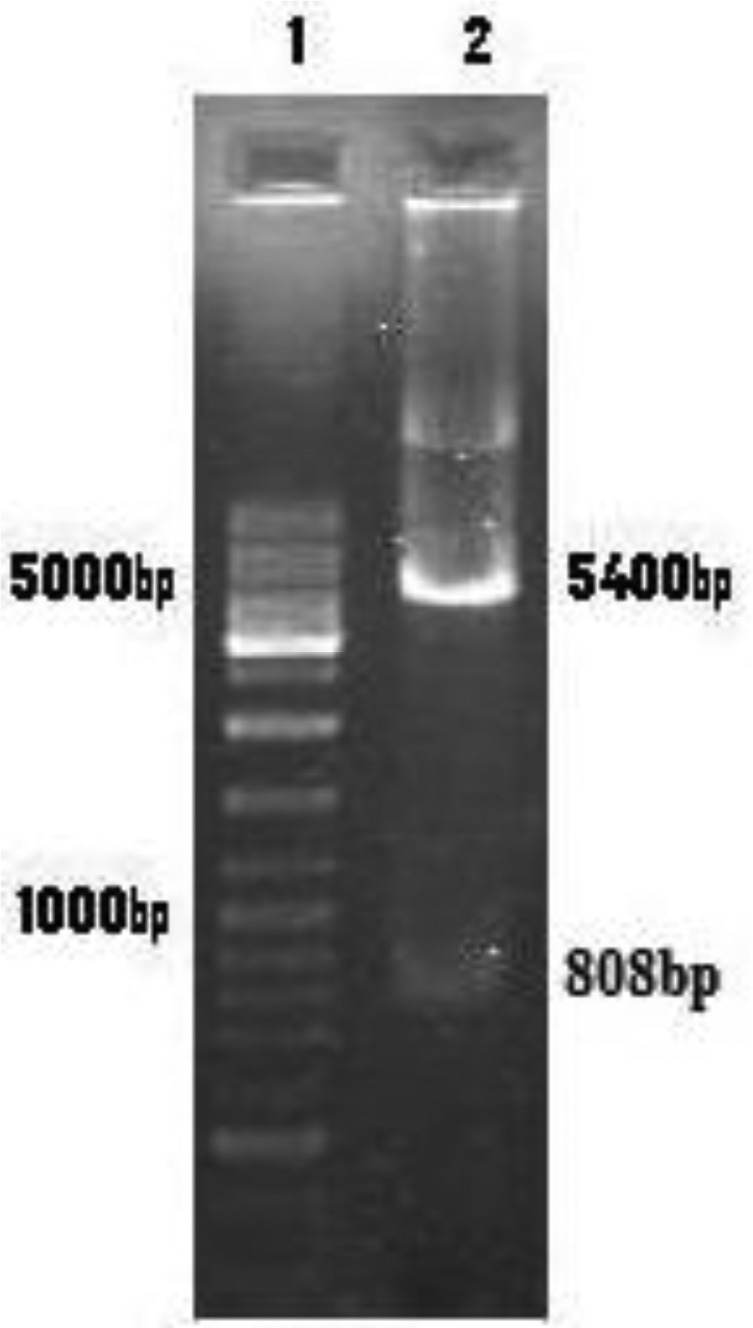
Identification of recombinant plasmid pcDNA-rPTR using enzyme digestion. Lane 1:100-bp DNA ladder marker. Lane 2: pcDNA-rPTR digested with KpnI and BamHI

*Leishmania major ptr1* gene was cloned into pBluescript, and digested with Bam HI and KpnI (Fig. 1), and subcloned into pcDNA3 as antisense (pcDNA-rPTR). To confirm the identity of the recombinant plasmid, pcDNA-rPTR was digested using restriction enzymes KpnI and BamHI and then an 808bp insert and a 5.4-kb vector fragment was observed using electrophoresis (Fig. 2).

### Promastigotes after transfection

Twenty-four hours after transfection, some dead promastigotes were observed. After selection with 40 µg/mL G418, untransfected promastigotes were dead, and transfected promastigotes were survived. The cell clones were isolated at 10-14 days after G418 selection.

### Inhibition of ptr1 expression by antisense in promastigotes

**Fig. 3:**
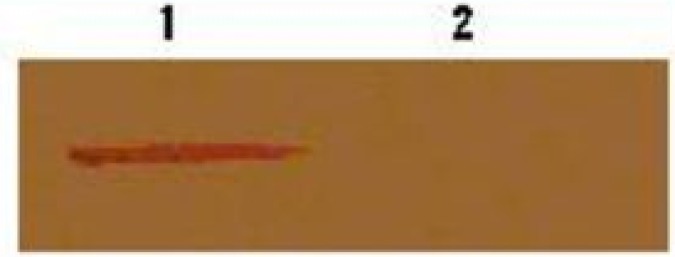
Western blot analysis to demonstrate lack of expression of PTR1 protein in antisense-transfected promastigotes Lane 1: Lysate of pcDNA3-transfected promastigotes Lane 2: Lysate of pcDNA-rPTR-transfected promastigotes

The PTR1 protein was expressed in pcDNA3-transfected promastigotes (Fig. 3, lane 1), but not expressed in pcDNA-rPTR–transfected promastigotes (Fig. 3, lane 2), indicating that the gene was inhibited by the antisense construct.

### Inhibition of ptr1 expression by antisense in amastigotes

**Fig. 4:**
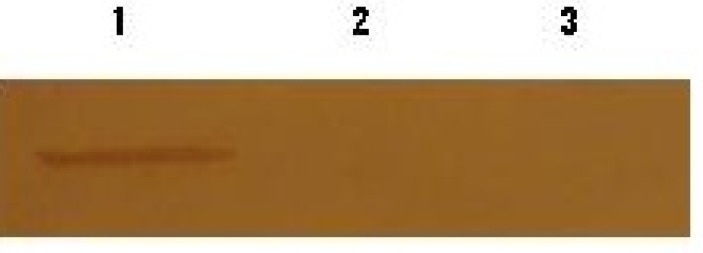
Analysis of expression protein by Western blotting Lane 1: Infected macrophages by amastigotes containing pcDNA3 /Lane 2: Extract macrophages Lane 3: Infected macrophages by amastigotes containing antisense (pcDNA-rPTR)

The results indicated that *ptr1* expression in amastigotes containing pcDNA- rPTR (antisense) was inhibited but inhibition was not seen in amastigotes containing pCDNA3 (Fig. 4).

## Discussion

The properties of *ptr1* suggest several mechanisms to explain how its overproduction creates MTX resistance. Because *ptr1* binds MTX tightly, it may sequester MTX from DHFR-TS (10). In addition, *ptr1* exhibits a broad specificity for pteridine substrates and reduces folate to the H2- and H4 forms (21). *Ptr1* may mediate MTX resistance through its ability to reduce folate to generate H2 folate, which is known to be extremely effective in relieving the inhibition of DHFR-TS by MTX in vitro (22, 23). Deletion of the *ptr1* gene is lethal to the promastigote in the presence of MTX (5). This gene has also been used to determine the species of *Leishmania* (24).

In this study, similar to others (25) pcDNA-rPTR was prepared and confirmed; the vector contains the *ptr1* gene oriented antiparallel to the sense.

*Leishmania major* promastigotes were transfected separately with pcDNA-rPTR and pcDNA3, and lysates were examined by western blot analysis, which showed that the expression of the *ptr1* gene was inhibited by pcDNA-rPTR successfully. The current data is similar to that generated by Chen et al., in which full-length antisense RNA was used to inhibit the expression of gp63 gene in *Leishmania amazonensis* (25). Although the targeted gene deletion in *L. major* GP63 was reported by others (15, 26), the present data demonstrated that inhibition occurs in the cytosol, as Dumas et al. reported in the study using cytosolic antisense RNA to regulate the expression of noncoding RNA in amastigotes (27). Small nucleolar RNA (snoRNA) genes might be silenced in *L. major*, *Leptomonas collosoma* and *Trypanosoma brucei* (28). Silencing is achieved in *Leptomonas collosoma* and *L. major* by expressing an antisense transcript complementary to the snoRNA gene, resulting in the accumulation of small interfering RNA (siRNA), the siRNA then eliminates the mature snoRNA (28). Other scientists have used antisense RNA to inhibit beta-tubulin synthesis in *Leishmania donovani* amastigote, as well as a mini-exon sequence to inhibit amastigote growth (29–33).

## Conclusion

In contrast to the previous studies, this work is the first report on inhibition of *Leishmania ptr1* by a full-length antisense construct. The results demonstrated that *ptr1* antisense RNA might be efficiently block *ptr1* mRNA and protein expression in *L. major* promastigotes and amastigotes which might be used as a model for gene inhibition of *Leishmania*. This approach might be used to study other infectious diseases and cancer cells when a single gene is involved.
